# MCIC: Automated Identification of Cellulases From Metagenomic Data and Characterization Based on Temperature and pH Dependence

**DOI:** 10.3389/fmicb.2020.567863

**Published:** 2020-10-23

**Authors:** Mehdi Foroozandeh Shahraki, Shohreh Ariaeenejad, Fereshteh Fallah Atanaki, Behrouz Zolfaghari, Takeshi Koshiba, Kaveh Kavousi, Ghasem Hosseini Salekdeh

**Affiliations:** ^1^Laboratory of Complex Biological Systems and Bioinformatics, Institute of Biochemistry and Biophysics, University of Tehran, Tehran, Iran; ^2^Department of Systems and Synthetic Biology, Agricultural Biotechnology Research Institute of Iran, Agricultural Research Education and Extension Organization, Karaj, Iran; ^3^Computer Science and Engineering Department, Indian Institute of Technology Guwahati, Guwahati, India; ^4^Department of Mathematics, Faculty of Education and Integrated Arts and Sciences, Waseda University, Tokyo, Japan; ^5^Department of Molecular Sciences, Macquarie University, Sydney, NSW, Australia

**Keywords:** cellulase, machine learning, metagenomics, enzyme screening, optimum temperature, optimum pH, MCIC

## Abstract

As the availability of high-throughput metagenomic data is increasing, agile and accurate tools are required to analyze and exploit this valuable and plentiful resource. Cellulose-degrading enzymes have various applications, and finding appropriate cellulases for different purposes is becoming increasingly challenging. An *in silico* screening method for high-throughput data can be of great assistance when combined with the characterization of thermal and pH dependence. By this means, various metagenomic sources with high cellulolytic potentials can be explored. Using a sequence similarity-based annotation and an ensemble of supervised learning algorithms, this study aims to identify and characterize cellulolytic enzymes from a given high-throughput metagenomic data based on optimum temperature and pH. The prediction performance of MCIC (metagenome cellulase identification and characterization) was evaluated through multiple iterations of sixfold cross-validation tests. This tool was also implemented for a comparative analysis of four metagenomic sources to estimate their cellulolytic profile and capabilities. For experimental validation of MCIC’s screening and prediction abilities, two identified enzymes from cattle rumen were subjected to cloning, expression, and characterization. To the best of our knowledge, this is the first time that a sequence-similarity based method is used alongside an ensemble machine learning model to identify and characterize cellulase enzymes from extensive metagenomic data. This study highlights the strength of machine learning techniques to predict enzymatic properties solely based on their sequence. MCIC is freely available as a python package and standalone toolkit for Windows and Linux-based operating systems with several functions to facilitate the screening and thermal and pH dependence prediction of cellulases.

## Introduction

Lignocellulosic biomass is a great potential resource for the production of bio-energy and bio-based material since it is largely abundant, inexpensive, and renewable (Demain et al., 2005). Cellulose, hemicellulose, and lignin together comprise the lignocellulosic biomass. Only a small volume of these biopolymers is currently used, with the rest being considered waste (Sánchez, 2009). Endoglucanases (EC 3.2.1.4), cellobiohydrolases or exoglucanases (EC 3.2.1.91), and beta-glucosidases (EC 3.2.1.21) are three major groups of cellulolytic enzymes and the process of cellulose degradation depends on their collaborative activity (Kumar et al., 2008). For many years, much attention has been paid to cellulases due to their potential of being utilized for various purposes including biofuel production, pulp, and paper, textile, food processing, animal feed, detergent, and agricultural industries as well as waste management (Kuhad et al., 2011). Evidently, each of these applications requires cellulases with specific characteristics and capabilities. The ability of enzymes to operate at a certain temperature or pH range is among the most critical attributes which make an enzyme suitable for a particular usage (Kirk et al., 2002).

Metagenomics is the culture-independent analysis of an environmental sample by a combination of molecular biology and genetics to extract, identify, characterize, and utilize almost all the genetic information embedded within that sample (Handelsman, 2005; Gharechahi and Salekdeh, 2018). The emergence of metagenomics has aided the isolation of various novel enzymes, cellulases included, from the uncultured microbiota of an environment. Unlike culture-dependent methods that are unable to present an inclusive understanding of microbial communities, their properties, and enzymatic capabilities, metagenomics offers a rich and valuable information resource to explore different environmental niches. Each microbial community has particularly evolved to meet the criteria of their ecosystem. As an instance, rumen microbiota has a rich hydrolyzing enzyme profile adapted to augment the digestion of the plant matter and plant-derived complex polysaccharides, such as cellulose, which dominate the ruminant diet (Stewart et al., 2019; Ariaeenejad et al., 2020c; Motahar et al., 2020). Termite has been known for its remarkable ability in the rapid deconstruction of recalcitrant woody biomass. This ability stems from complex relationships among termite intestinal symbionts (Liu et al., 2019).

Numerous enzymes have been isolated from metagenomic samples without the employment of computational methods including some thermophilic cellulases that were derived from environments such as biogas digester, sugarcane bagasse, rice straw compost, and hot springs (Geng et al., 2012; Yeh et al., 2013; Schröder et al., 2014; Kanokratana et al., 2015). However, the process of mining for novel enzymes solely through functional screening techniques and without the utilization of *in silico* methods can be laborious and time-consuming. For instance, to isolate highly thermostable beta-xylosidases from hot spring soil microbiota, the researchers had to express, examine, and screen 269 candidate proteins based on their xylosidase activity (Sato et al., 2017). Doubtlessly, powerful *in silico* approaches could effectively facilitate this process and reduce experimental expenses and time consumption.

As the availability of metagenomic data is rapidly growing, due to the advent of next-generation sequencing technologies and their constant advancements, employing computational methods instead of wet-lab experiments to identify new enzymes with specific properties, can significantly reduce the costs and accelerate the process. In this context, several sequence-based enzyme analysis tools have been developed and extensively used in recent years. These tools are designed to tackle several key problems in this field such as *in silico* prediction of enzymatic functions (Dalkiran et al., 2018; Li et al., 2018; Zhang et al., 2020), protein structure homology-modeling (Waterhouse et al., 2018) and docking (Grosdidier et al., 2011), etc. Moreover, some studies have been dedicated to the sequence-based prediction of thermostability (Zahiri, 2016), and computational engineering of enzymes toward specific characteristics of interest (Mazurenko et al., 2020).

One of the major computational approaches that has been rigorously employed in bioinformatics is machine learning, which as the literature can prove, is capable of mapping the relationships between the primary structure of proteins and their different properties and make reasonable predictions based upon them (Shastry and Sanjay, 2020). Machine learning techniques have been successfully applied so predict various properties of proteins such as activity (Ariaeenejad et al., 2018), tertiary structure (Cheng et al., 2008), subcellular localization (Almagro Armenteros et al., 2017), stability at different environmental conditions (Wu et al., 2009), etc. More specifically, Yan and Wu (2012) used a neural network to predict optimum pH and temperature of endoglucanases (EC 3.2.1.4) from their primary structure. AcalPred is another study that utilizes support vector machines to discriminate between acidic and alkaline enzymes based on their amino acid sequences (Lin et al., 2013). Moreover, several research studies have focused on the sequence-based prediction of protein thermostability (Ebrahimi and Ebrahimie, 2010; Pucci et al., 2014). In a 2019 study, Li et al. (2019) utilized machine learning to predict the optimum growth temperature (OGT) for microorganism based in their proteome and subsequently, used the predicted OGT alongside with the enzymes’ amino acid compositions to predict their catalytic optima. In another research, Ariaeenejad et al. (2018) applied a regression model based on pseudo amino acid composition to predict the optimum temperature and pH of xylanase in strains of *Bacillus subtilis* enzymes.

Herein, we present an automated pipeline for sequence-based identification of cellulose-degrading enzymes, as well as a machine learning approach aimed at the prediction of their thermal and pH dependence. MCIC (metagenome cellulase identification and characterization) can explore metagenomic assembled contigs and screens them to find probable cellulolytic enzymes and classifies them based on their optimum pH and temperature. Furthermore, four metagenomic data from the soil, termite gut, sheep, and cattle rumen sources were analyzed by MCIC and their cellulolytic profiles are compared. Two computationally predicted enzymes from cattle rumen data were cloned, expressed, and tested to validate the tool’s competence.

## Materials and Methods

### Development and Evaluation of the Prediction Model

#### Dataset Preparation

**FIGURE 1:**
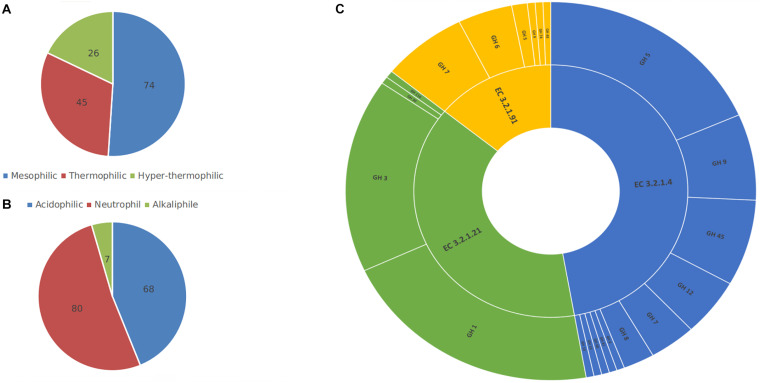
Samples in temperature **(A)** and pH **(B)** datasets were divided into three classes based on their reported optima. The non-redundant union **(C)** of both datasets, had 163 instances with 3 different EC numbers and from 16 different glycoside hydrolase (GH) families. This figure shows the composition of each class or family in the collected datasets.

The first step toward training a model is data collection and since there was not any previous research on the prediction of thermal and pH dependence of three enzyme families involved in cellulose degradation, two new datasets for temperature and pH optima had to be collected. The BRENDA (Jeske et al., 2019) and UniProt (Bateman, 2019) databases were explored for enzyme families with EC 3.2.1.4, EC 3.2.1.21, and EC 3.2.1.91 and samples with reported optima were extracted. Since the existence of any character that does not represent an amino acid residue will interfere with the process of feature generation and learning, therefore any character other than amino acid symbols were removed from extracted sequences. Redundant or highly similar enzymes’ were removed using the CD-Hit tool with a cut-off value of 0.9 (Huang et al., 2010). To reduce the redundancy, CD-Hit clusters highly homologous sequences and keeps one sample from each cluster. The final datasets consisted of 155 and 145 samples with optimum pH and temperature labels, respectively, and the non-redundant union of both datasets had 163 instances. Samples were labeled according to their reported optima. For temperature dataset, samples were labeled into mesophilic (T_opt_ < 50°C), thermophilic (50°C < T_opt_ < 75°C) and hyper-thermophilic (75°C < T_opt_). Likewise, samples in the pH dataset were labeled into acidic (pH_opt_ < 5), neutral (5 < pH_opt_ < 8), and alkaline (8 < pH_opt_). Figure 1 presents the ratio of enzyme families, and classes in the datasets.

Data imbalance is a common obstacle in many classification problems. As illustrated in Figure 1, the collected datasets are relatively imbalanced. More severely, pH dataset lacks sufficient numbers of alkaline cellulase samples, with seven alkaline instances comprising a ratio of less than five percent of the whole dataset. This insufficiency of samples leads to decreased ability of the final prediction model in detecting alkaline cellulases. In order to address the data imbalance problem, synthetic minority over-sampling technique (SMOTE) was implemented on training datasets (Chawla et al., 2002). SMOTE tackles this problem by adding new synthetic instances of the minority classes, to the point where it is in balance with other majority classes.

#### Feature Generation

Utilization of a proper set of features is one of the most critical steps in the effort to train an accurate predictor. Due to the lack of precise evidence on the most related protein features to its thermal and pH dependence, in this study, numerous protein features were calculated and selected in order to find the most appropriate set of features for the current classification problem. In the feature generation step, we utilized iFeature and Pfeature that are two publicly available tools capable of generating a comprehensive spectrum of descriptors to facilitate the numerical representation of biological sequences for machine learning purposes (Chen et al., 2018; Pande et al., 2019). These sequence-based and length-independent features incorporate various properties of proteins such as composition of atoms, bonds, amino acids, and dipeptides as well as physico-chemical properties, repeats, distribution, etc. Many of the generated descriptors were duplicates and thus were removed. Supplementary Table 1 presents a summary of computed protein features and their dimensionality after removing duplicate descriptors.

Consequently, a feature vector with a dimension of 6524 unique descriptors was generated for each enzyme sample in the datasets. Due to the fact that different descriptors have dissimilar ranges, all raw values had to be transformed into the same scale. MinMaxScaler method was employed to rescale features’ value ranges between 0 and 1.

#### Feature and Model Selection

Enzymatic attributes, including temperature and pH dependence, are under the influence of various sequential, structural, and physiochemical features; nonetheless, not every generated descriptor is equally related to the attributes of interest. Therefore, a process of selecting the most relevant features to the optimum pH and temperature was required to prune features with lower importance and predictive ability. Filter feature selection methods use a statistical measure to score and rank features. For this step, Chi-square and ANOVA *F*-test were tested, and due to better results, the *F*-test was finally applied as the filter method, and the best descriptors were nominated by the SelectKBest method, with K-values of 253 and 93 for temperature and pH models, respectively (number of features with *P*-value < 0.05). Since multiple tests are being performed, Benjamini–Hochberg (Ferreira and Zwinderman, 2006) method was used as a false discovery rate controlling procedure to obtain realistic *P*-values in order to find the number of features with significant relationship with modeled responses.

Since there are numerous classification methods with different strengths and weaknesses, we had to find those that are most suited for this problem. Various classifiers were tested including, Multilayer perceptron (MLP), Decision Tree, Random Forest (RF), NaÏve Bayes, Gaussian Process, Bagging Classifier, AdaBoost, K-Nearest Neighbors, Support Vector Machine (SVM), XGBoost, and Gradient Boosting, all of which were implemented from sci-kit learn python package (Pedregosa et al., 2011). Among these methods, MLP, SVM, and RF depicted considerably better prediction performance. MLPs are a subset of artificial neural networks (ANN) that are inspired by the structure and function of actual biological neural networks and are widely used supervised learning algorithms (Tadeusiewicz, 1995). RF is consisted of multiple decision trees which result in reduced variance and better generalization in comparison to single decision trees (Breiman, 2001). For classification problems, support vector machines construct hyperplanes using a variety of kernel functions (Cortes and Vapnik, 1995). RF and SVM are popular methods in computational biology due to their ability in dealing with high-dimensional feature space, small number of samples, and complex data structures (Ben-Hur et al., 2008; Qi, 2012).

After testing various combinations of different classification algorithms, an ensemble classifier was built from a MLP (with two hidden layers, 200 nodes in each layer, ReLU as activation function, and Adam optimizer), a RF (with 200 decision trees and information gain as splitting criteria), and a SVM (with radial basis function kernel). The ensemble method decides the final output from weighted soft voting between three mentioned classifiers. In the process of soft voting, each model returns an array representing its computed probability of occurrence for each class.

The workflow mentioned above required several hyper-parameter tuning steps, all of which were performed using the GridSearchCV method (Yu and Zhu, 2020). There are several approaches to detect the best combination of hyperparameters for a machine learning model. The GridSearchCV is an algorithm which given multiple options for each hyper-parameter, tries every possible combination to build the desired model and evaluates that model through cross-validation tests. This method can thoroughly investigate the hyper-parameter space to find the best configuration for the model which achieves the best performance.

#### Evaluation

Among evaluation strategies for machine learning models, multiple iterations of random train-test splitting and single cross-validation tests are two most commonly used approaches. In this study, for the purpose of evaluation, 100 iterations of sixfold cross-validation tests with different random seeds were performed to assure a thorough assessment of the prediction models by training and testing them on various random combinations of data. In the sixfold cross-validation, the dataset was randomly split into six equal subsamples, five of which were used as training sets that were subjected to feature selection, oversampling, and one subsample was then used for testing with different evaluation metrics. This validation is executed six times leaving out one subsample each time as the test set to ensure that all samples in the set were tested.

Accuracy, macro-recall, macro-precision, macro-f1 scores are among the most commonly used metrics and are calculated through the following formulae:

(1) $$\text{Accuracy}=\frac{\text{TP}+\text{TN}}{\text{TP}+\text{FP}+\text{TN}+\text{FN}}$$

(2) $$\text{Recall}=\frac{\text{TP}}{\text{TP}+\text{FN}}$$

(3) $$\text{Precision}=\frac{\text{TN}}{\text{TN}+\text{FP}}$$

(4) $$\text{F}\,1=2.\frac{\text{Precision}\times \text{Recall}}{\text{Precision}+\text{Recall}}$$

**FIGURE 2:**
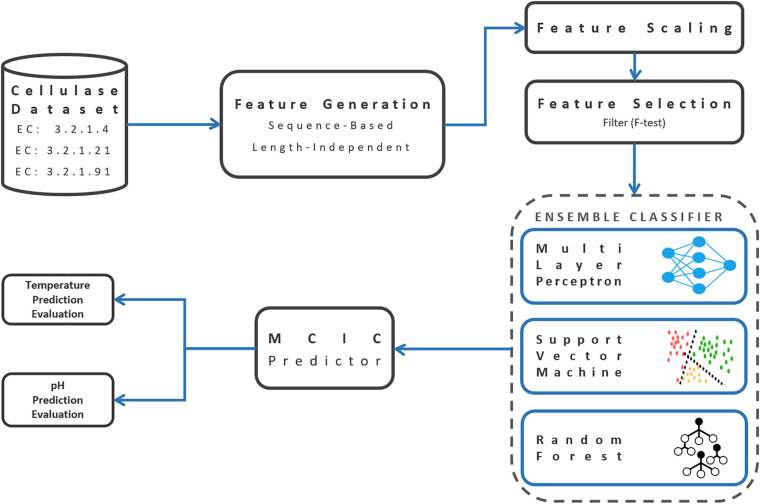
Schematic workflow of building the MCIC’s prediction models.

Here, TP (true positive) and TN (true negative) are positive and negative examples, respectively, that were correctly predicted. Accordingly, FP (false positive) and FN (false negative) were mistakenly classified. “macro” prefix refers to the unweighted average of each metric among different classes. Sci-kit learn python package was used several times during the above mentioned pipeline of development and evaluation of the prediction model (Pedregosa et al., 2011). Figure 2 illustrates the graphical workflow of developing MCIC’s prediction models.

### Comparative Metagenome Analysis

#### Assembly of Metagenomic Data

Firstly, raw data was prepared for samples in four different environments, including termite gut, soil, sheep rumen, and cattle rumen. Alongside a cattle rumen raw reads that was obtained from authors’ another study (Gharechahi et al., 2020), three other samples were downloaded from NCBI’s Short Read Archive (SRA) including termite sample (Whole genome shotgun sequencing of *Macrotermes natalensis* soldier gut metagenome) under accession number: SRR797686 (Hu et al., 2019), soil sample raw reads downloaded under accession number: ERR1939274 (Orellana et al., 2018), and sheep rumen with accession number SRR1222429 (Kamke et al., 2016). Reads quality was checked using FastQC tools, and reads with relatively poor quality (Phred score < 20) were discarded for further analysis. The subsequent cleaned reads were given to MEGAHIT as inputs (Li et al., 2015). MEGAHIT is a standalone software available as both CPU and GPU versions. This tool allows users to perform large and complex metagenomics assembly up to hundreds of GB data. Metagenome assembly was carried out using options - -kmin-1pass, - -k-list 27,37,47,57,67,77,87, - -m 60e + 10, - -min-contig-len 300, -t 16. The results from *de novo* assembly of metagenomic data contained millions of assigned contigs.

#### Identification and Characterization of Cellulases From Assembled Contigs

In order to identify putative cellulase genes among assembled contigs, the sequence similarity-based method of NCBI’s BLASTx (Altschul et al., 1990) was executed against the cellulase sequences dataset (163 samples), which were used to train the prediction machine. These 163 sequences are the non-redundant union of both pH and temperature datasets. The BLAST results were then filtered on the basis of bit-score (Pearson, 2013). The minimum bit-score cut-off was chosen to be 50 which indicates homology with the known cellulase samples. The screened cellulase genes are then translated and the resulting amino acid sequences are then given to the prediction module as inputs to be characterized in terms of thermal and pH dependence.

#### Identification, Cloning, Expression, Purification, and Characterization of Two Cellulolytic Enzymes’ Genes

With the objective of experimental validation of MCIC’s predictive performance and demonstration of its potential applicability for mining metagenomic big data for cellulases, MCIC was exploited to computationally identify and characterize putative cellulases within cattle rumen’s metagenomic data. As the process of gene cloning and production of enzymes has lower success-rate when the abundance of the target gene in the sample is not high, and additionally since this process is costly and time-consuming, therefore some strict measures were taken to refine the final list of candidates as much as possible. Two main criteria were considered for selecting the final set of target candidates for further experiments. First, by mapping reads back to assembled contigs using the BWA (Li, 2013), only contigs that were in the top 25% of the mapped reads were retained. Bit-scores assigned to each computationally identified cellulase were considered as the secondary selection filter, and sequences with lower than 300 bit-scores were removed. The remaining putative cellulase genes in the filtered shortlist were nominated for cloning, expression and further experiments in order to verify the MCIC’s ability in correct cellulase identification and characterization. These wet-lab analysis were aimed at experimental validation of MCIC’s predictive performance.

In order to acquire cellulase genes, metagenomic DNA templates from cattle rumen were used for polymerase chain reaction (PCR) amplification with two pairs of primers. For PersiCel5 a forward (5′-TAATAGGCTAGCATGAAGAAGTC CTTTGTATTTGT-3′) primer with NheI restriction site and a reverse (5′-TGATAGGTCGACTTATTTTATATCTATCTCATT GCG-3′) primers with SalI restriction site was used for amplification. Similarly, PersiCel6 was PCR-amplified using a forward (5′-TAATAGGCTAGCATGAATAAGAAGCATTTGC GG-3′) primer with NheI site and reverse (5′-TGATAGGCG GCCGCCTATTTTCCAGCCTTCTCCT-3′) primers containing NotI restriction site. The resulting PCR products were detected on agarose gel 1.5% (w/v) and purified using the gel extraction kit (BioRon, Germany). Purified DNA fragments were cloned and digested into the pET28a.

The resulting plasmids were then transformed into the *Escherichia coli* BL21 (DE3) and correct insertion was confirmed by sequencing. In the Luria-Bertoni (LB) medium, the recombinant strain pET28a was cultivated at the temperature of 37°C. Adding isopropyl-β-D-thiogalactopyranoside (IPTG) to a final concentration of 0.4 mM for 20 h at 20°C, expression of enzymes was induced. By utilizing Ni-NTA Fast Start Kit (Qiagen, Hilden, Germany), N-terminal Histidine-tagged recombinant protein was purified and evaluated by sodium dodecyl sulfate-polyacrylamide gel electrophoresis (SDS-PAGE).

Two candidate enzymes produced from successful cloning, expression, and purification were named PersiCel5 and PersiCel6. Both enzymes were subjected to further biophysical experiments. Nucleotide sequences of both PersiCel5 and PersiCel6 were submitted to GenBank and are available in the Supplementary Material. Protein concentrations were determined through the Bradford method using the bovine serum albumin as the standard. By measuring the optical density (OD) of chromatography eluent at 280 nm, the protein concentration was estimated.

To determine the optimum pH of enzyme activity, 10 mM phosphate buffer was prepared at different pH (4–11), and after added enzyme was incubated with a substrate for 20 min at room temperature. The DNS was used to measure activity. In order to determine the optimum temperature of enzyme activity, enzyme solution in 10 mM phosphate buffer (pH 8) with substrate was incubated in the different temperature (30–90°C) for 20 min and DNS was used to measure activity (Ariaeenejad et al., 2019, 2020b,a). For reporting, relative activity was considered as percentage of the highest activity.

## Results and Discussion

### Prediction Model’s Performance

Generated protein descriptors incorporate various molecular and sequential information with different degrees of relevance to the enzymes’ thermal or pH dependence. With the purpose of better illustration, the overall importance scores of different features, at the level of feature category, were calculated and shown in Supplementary Figure 1. The feature category importance score is calculated as the average of all F-scores corresponding to single descriptors in that particular feature category.

**FIGURE 3:**
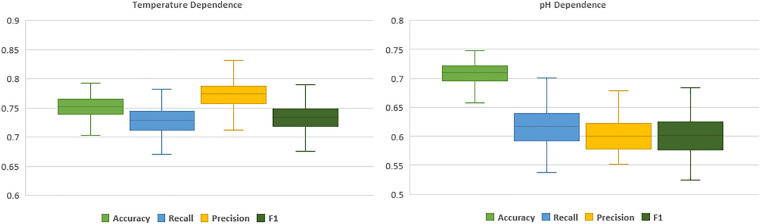
Boxplots of temperature (left) and pH (right) dependence prediction performance computed through 100 sixfold CV tests.

Even though models demonstrated agreement upon correct predictions, in case of mis-classification their outputs were mostly different. This diversity in predictions stems from the dissimilarity in the basis of these classification algorithms. Hence, employing an ensemble method such as a voting classifier, could make us capable of exploiting three different machine learning approaches for a single task of prediction. The ensemble voting classifier, enhanced the overall performance of the model compared to single classifiers therefore it was the method of choice. Reported performance metrics are computed through 100 iterations of sixfold cross-validation tests. In each CV iteration, to assure the unbiased and effective evaluation, datasets were shuffled with different random seeds before splitting. Figure 3 and Table 1 represent the evaluation results of final model through 100 iterations sixfold CV tests.

**TABLE 1:** MCIC’s prediction performance during 100 iterations of sixfold CV tests.

	Accuracy		Macro-recall		Macro-precision		Macro-F1	
	T_opt_	pH_opt_	T_opt_	pH_opt_	T_opt_	pH_opt_	T_opt_	pH_opt_
CV performance	0.75	0.71	0.73	0.62	0.77	0.60	0.73	0.60

To elaborate, in the prediction of temperature dependence, the model showed noticeably higher recall score (∼0.84) in the hyper-thermophilic class, in comparison to mesophilic and moderate-thermophilic classes (∼0.79 and ∼0.67, respectively), which suggest better applicability of this model in *in silico* characterization of hyper-thermophilic cellulases. In the prediction of cellulases’ pH dependence, while the model had higher than 0.7 recall score in both acidic and neutral categories, it depicted a weak predictive performance in alkaline category (∼0.28). This poor performance on prediction of the alkaline cellulases is resulting from very small number of samples (seven) and lack of enough instances in the training dataset that was collected from the available cellulases in the literature with reported pH and temperature optima. Moreover, the relatively low variation in overall performance and the small error bars in the boxplots, imply the robustness of the models in the evaluation through 100 iterations of sixfold cross-validation test.

### Comparative Metagenomic Analysis of Cellulolytic Profiles From Different Sources

After the assembly of raw metagenomic data for all four samples, millions of contigs were acquired. The total number of contigs obtained from *de novo* assembly of high-quality clean reads in each sample is presented in Supplementary Table 2. It was anticipated that undoubtedly copious enzyme genes were among these contigs. Therefore, a BLASTx against our dataset of cellulases could address the challenge of identifying cellulolytic enzymes. Numbers of identified cellulases from each metagenome source and the ratio of cellulases to all assembled contigs are also presented at Supplementary Table 2. After identification and translation of target cellulase genes, amino-acid sequences were used as inputs to the prediction tool for determination of thermal and pH dependence. Rapid and accurate analysis of the prevalence, pH, and temperature dependence of cellulose degrading enzymes found within a sample can be considered a significant step to obtain a comprehensive understanding of cellulolytic profile and abilities of different environments. Supplementary Figure 2 illustrates the detailed results of the comparative analysis of four metagenome samples by using MCIC.

The comparative analysis results indicate that the sheep rumen had the highest number of cellulase genes while the ratio of cellulases to all assembled contigs was higher in cattle rumen. In comparison, fewer cellulases were found within soil and termite samples. The significantly higher abundance of cellulolytic enzymes in the rumen environments proves the cellulolytic abilities of ruminants’ digestive system (Stewart et al., 2019). Not surprisingly, the majority of identified cellulases in all four samples were neutral and mesophilic. However, comparatively, the soil environmental sample had more acidic enzymes.

### Identified, Cloned, Expressed, and Characterized Cellulases

Aiming to experimentally validate the MCIC’s ability to facilitate the process of targeted mining for cellulases in metagenomic libraries, this tool was utilized to isolate and produce two novel cellulases from cattle rumen metagenome. After applying a two-step filtration, based on number of mapped reads and the bit-score, a shortlist of computationally identified candidates were obtained to be subjected to further experimental analysis. Consequently, two novel cellulases named PersiCel5 and PersiCel6 were produced and characterize through wet-lab experiments and both enzymes are used for other studies on enhancing lignocellulose degradation. Table 2 presents the predicted classes of thermal and pH dependence for two enzymes in addition to the real values of pH and temperature optima.

**TABLE 2:** Two novel cellulolytic enzymes were discovered from camel rumen metagenome and are being investigated in other studies. This table represents the detected enzymatic function of each enzyme and the comparison of predicted dependence characteristics with real pH and temperature optima values. MCIC was able to correctly predict the desired attributes.

Enzyme name	Enzymatic function	Predicted thermal dependence	Predicted pH dependence	Optimum temperature	Optimum pH
PersiCel5	Endo-glucanase	Mesophilic	Neutral	50	6.5
PersiCel6	Endo-glucanase	Thermophilic	Neutral	70	7.5

The successful results for the targeted enzyme mining by the help of MCIC and its predictions indicate the potential applicability of this tool for a better exploration of different metagenomic data. To the best of our knowledge, this is the first study that provides software and descriptions of an automated pipeline for computational identification and pH and temperature dependence characterization of cellulose-degrading enzymes from high-throughput data and its ability was verified by wet-lab experiments and comparative analysis of different metagenome samples. As generally developing powerful machine learning-based models requires large training datasets, one of the greatest challenges of this study was the relatively limited number of cellulases existing in public databases with reported temperature and pH optima. Therefore, to compensate for the relatively small size of training datasets, in a very time-consuming and computationally intensive stage of this study, several classification algorithms with various hyper-parameters were rigorously explored, both individually and in different combinations, to find the optimal configuration with best performance. Moreover, detailed analysis of the classification results suggested that, despite the utilization of SMOTE and its contribution in over-sampling of the minority classes, the models’ performances were generally higher in prediction of classes with more training data in comparison to ones with fewer training instances. This implies that in future studies, when more experimentally identified and characterized cellulase enzymes are available, development of more accurate machine learning-based models for this task will be possible. Undoubtedly, this methodology can be generalized to other enzymatic attributes and for other enzyme families. Through the implementation of such automated pipelines with various predictive models, the challenge of targeted enzyme identification as well as agile, effective, and inexpensive screening of high-throughput data can be effectively addressed. By this means, the number of potential candidate enzymes with specific characteristics of interest could be significantly reduced prior to engaging the wet-lab experiments. From the prediction perspective, unlike Yan and Wu (2012) study which was only capable of performing the prediction for endoglucanases (EC 3.2.1.4), our method could extend the ability to other cellulolytic-enzymes cellobiohydrolases (EC 3.2.1.91), and beta-glucosidases (EC 3.2.1.21).

### MCIC Standalone Toolkit

Metagenome cellulase identification and characterization is freely accessible in the form of a standalone toolkit and a python package. This tool helps users with screening of high-throughput metagenomic contigs in order to find probable cellulases and classify them on the basis of their pH and thermal dependence. Furthermore, both MCIC’s prediction and screening tools are individually operational. The predictor accepts single and multiple cellulases in form of amino-acid sequence, while the screening tool, can identify and screen cellulases among millions of metagenome-derived contigs. The standalone software is available for Windows and Linux-based operating systems. MCIC is downloadable from https://cbb.ut.ac.ir/MCIC, and MCIC’s repository on GitHub^1^. Further description and help about different functions of MCIC and their usage is available in the tool’s manual.

The MCIC software is particularly aimed at facilitating and automation of the process for getting access to cellulolytic enzymes existing in extensive metagenomic data in a targeted manner. As the main criterion for identification of cellulase-coding genes, the bit-score cut-off, is configurable by the users therefore, if this criterion is set more strictly to higher values, only sequences with more similarity to sample sequences in the reference database will be retained. This way, although potentially numerous cellulases with less sequence-similarity to the database may be omitted, users can obtain more accurate predictions of pH and temperature dependence due to the similarity of identified sequences to the predictive models’ training datasets.

## Conclusion

In this study, MCIC tool was designed and implemented in order to address two major objectives, one being the identification of probable cellulose degrading enzymes among metagenomic contigs and the other being the sequence-based characterization of their temperature and pH dependence. For the first purpose, a sequence similarity-based method, was employed to screen cellulase enzymes and for the prediction task, an ensemble of three supervised learning classifiers, MLP, RF, and SVM, were trained by a combination and selection of various sequence-based descriptors to classify cellulases on the basis of their temperature and pH dependence.

Moreover, MCIC was used for the analysis and comparison of cellulolytic profiles of four metagenome samples. This comparative analysis highlighted the extended enzymatic diversity and cellulose-hydrolytic ability of ruminants’ rumen microbial community. Two identified cellulases were cloned, produced, and experimentally tested in order to validate the tool’s abilities. The results of this study imply the competence of both machine learning techniques and sequence-based protein features for the prediction of enzymatic attributes. The MCIC toolkit has been made available as a standalone software and a python package and offers a variety of services. Since a plethora of enzymes are in constant demand and discovery of novel enzymes for various purposes is of great importance, similar analytic and predictive tools are needed for other enzyme families and this may be a possible direction for future studies. Moreover, different machine learning techniques are being developed and advanced and they have proved effective in solving various biological problems.

## Data Availability Statement

Publicly available datasets were analyzed in this study. This data can be found here: accession number SRR797686, https://www.ebi.ac.uk/biosamples/samples/SAMN08019258; accession number ERR1939274, https://trace.ncbi.nlm.nih.gov/Traces/sra/?run=ERR1939274; accession number SRR1222429, https://www.ebi.ac.uk/biosamples/samples/SAMN06452595.

## Author Contributions

MFS performed software development, evaluation, implementation, and comparative analysis. KK performed the theoretical design and provided significant advice on the method design. FFA performed the acquisition and assembly of metagenomic data and assisted in software development. SA executed the gene cloning, expression, purification, and biochemical characterization. BZ helped to implement the packages, data analysis, review, and editing. TK performed complete review, redesign, and check the machine learning models and pipeline according to the reviewers’ comments, writing, review, and editing. GHS supervised the study and revised the manuscript. All authors read and approved the final manuscript.

## Conflict of Interest

The authors declare that the research was conducted in the absence of any commercial or financial relationships that could be construed as a potential conflict of interest.

## Footnotes

1 https://github.com/mehdiforoozandeh/MCIC
