# Supplementary material for: MCIC: Automated Identification of Cellulases From Metagenomic Data and Characterization Based on Temperature and pH Dependence
Source: Front Microbiol. 2020 Oct 23;11:567863. doi: 10.3389/fmicb.2020.567863 (PMC7645119; doi:10.3389/fmicb.2020.567863)
Supplement: Supplementary file 1 [file Data_Sheet_1.docx]

**Supplementary Material**

1. **Feature Importance Analysis**

| **Features** | **Dimension** |
| --- | --- |
| Composition of k-spaced Amino Acid Pairs | 2400 |
| Conjoint k-spaced triad (Shen et al., 2007) | 2040 |
| Dipeptide Deviation from Expected Mean | 400 |
| Geary Autocorrelation | 240 |
| Moran Autocorrelation | 240 |
| Normalized Moreau-Broto Autocorrelation | 240 |
| CTD (Distribution) | 180 |
| Composition of k-spaced amino acid group pairs | 150 |
| Grouped Tripeptide Composition | 125 |
| Quasi-Sequence-Order | 100 |
| Amphiphilic Pseudo-Amino Acid Composition (Chou, 2005) | 80 |
| Sequence-Order-Coupling number | 60 |
| Pseudo-Amino Acid Composition (Shen and Chou, 2008) | 50 |
| CTD (Transition) | 39 |
| CTD (Composition) | 38 |
| Physico-Chemical Properties | 27 |
| Shannon Entropy (Properties) | 21 |
| Residual Repeats | 20 |
| Distribution of Residues | 20 |
| Shannon Entropy (Residue Level) | 20 |
| Amino Acid Composition | 20 |
| Atoms Composition | 5 |
| Grouped amino acid composition | 5 |
| Bonds Composition | 3 |
| Shannon Entropy (Protein Level) | 1 |

Supplementary Table 1: For each enzyme sample in the datasets various protein features were computed. This table presents different feature groups and their corresponding dimensionality after the removal of duplicate descriptors.

With the purpose of better illustration, the overall importance scores of different features, at the level of feature category, were calculated and shown in Supplementary Figure 1. The feature category importance score is calculated as the average of all F-scores corresponding to single descriptors in that particular feature category. The feature importance analysis was not a part of the workflow that was used for the development of this tool and it was conducted independently from feature selection, aiming for descriptive purposes only to provide readers with a brief overview of the predictive information embedded within each feature category.

**
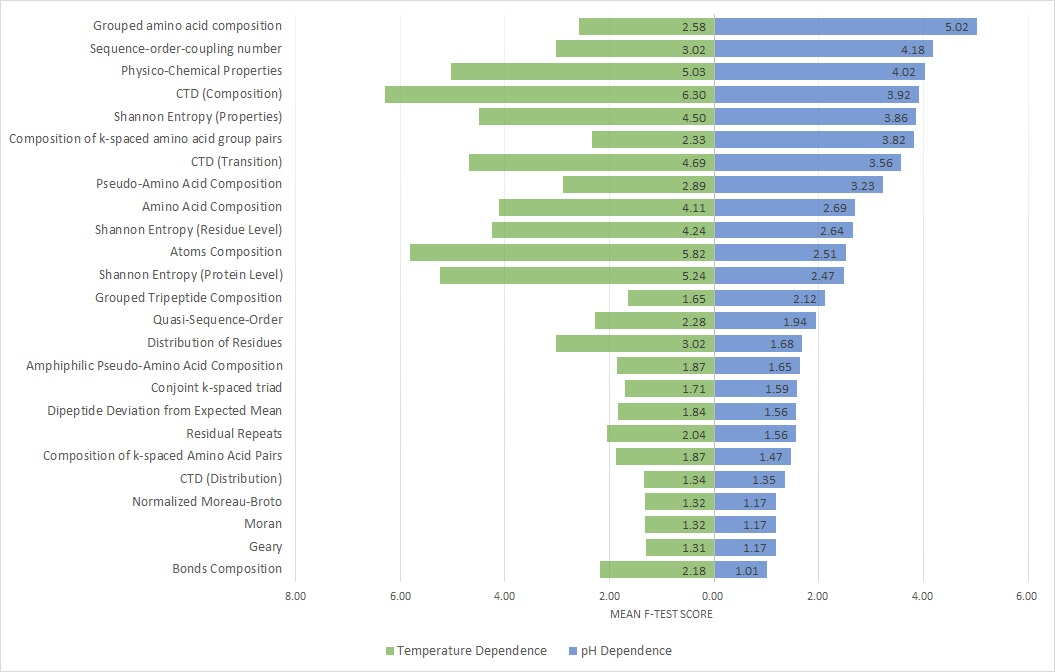
**

Supplementary Figure 1 This figure illustrates the importance of each feature set for the prediction of pH and temperature dependence. Detailed information about each feature group and their calculation formulas are completely elaborated in iFeature (Chen et al., 2018) and PFeature (Pande et al., 2019) tool manuals.

As presented in Supplementary Figure 1, CTD (Composition), physico-chemical properties, atoms composition and Shannon Entropy are among the more important features. When considered separately, the features that are more important to temperature dependence are not necessarily equally relevant to pH dependence which implies the contrasting molecular basis of these attributes.

1. **Comparative Metagenomic Analysis**

**
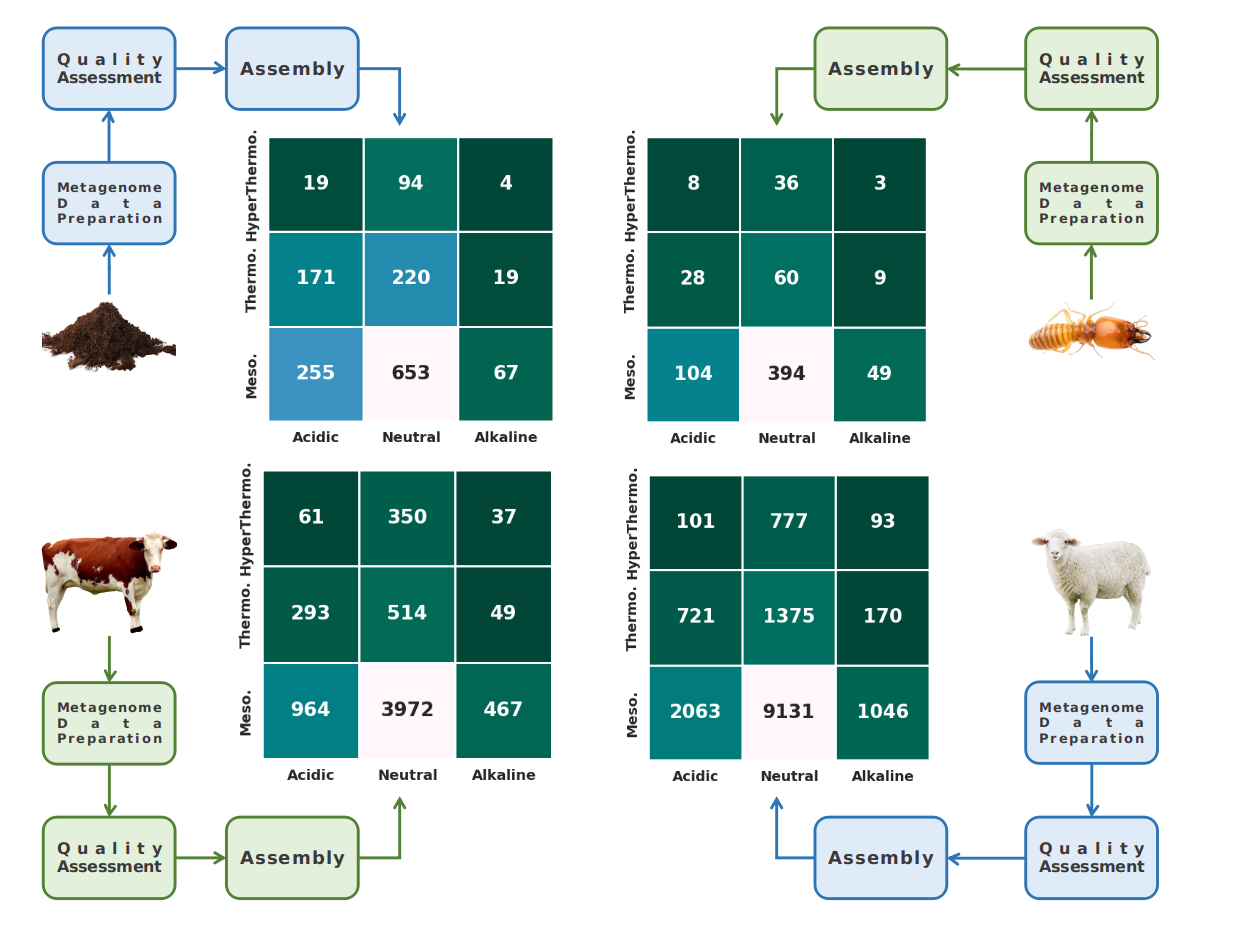
**

Supplementary Figure 2: An overview of metagenome comparative analysis of four different samples. Heat-maps represent the number of cellulases found in 9 possible classes of pH and temperature dependence.

|  | **Total No. of contigs** | **No. of identified cellulases** | **Cellulase / Contig** |
| --- | --- | --- | --- |
| **Cattle rumen** | 1,623,558 | 6707 | 0.0041 |
| **Sheep rumen** | 4,185,018 | 15477 | 0.0036 |
| **Termite gut** | 972,941 | 691 | 0.0010 |
| **Soil** | 1,479,331 | 1502 | 0.00071 |

Supplementary Table 2**:** This table presents the number of obtained contigs from the assembly of raw metagenome data in addition to the number of cellulases that were identified from each sample. The cellulase/contig ration represents the prevalence of cellulose-degrading enzymes in each environment.

The comparative analysis results indicate that the sheep rumen had the highest number of cellulase genes while the ratio of cellulases to all assembled contigs was higher in cattle rumen. In comparison, fewer cellulases were found within soil and termite samples. The significantly higher abundance of cellulolytic enzymes in the rumen environments proves the cellulolytic abilities of ruminants’ digestive system (Stewart et al., 2019). Not surprisingly, the majority of identified cellulases in all four samples were neutral and mesophilic. However, comparatively, the soil environmental sample had more acidic enzymes.

1. **Sequence information of PersiCel5 and PersiCel6**

The provisional accession IDs are MN821011 and MN821012 for PersiCel5 and PersiCel6 respectively.

- 1. **PersiCel5**

LOCUS BSeq#1 1026 bp DNA linear ENV 14-OCT-2019

ACCESSION MN821011 (will be available from 2021)

ORGANISM uncultured bacterium

Bacteria; environmental samples.

gene 1..1026

/gene="PersiCel5"

CDS 1..1026

/gene="PersiCel5"

/codon_start=1

/transl_table=11

/product="PersiCel5"

/translation= "MKKSFVFVLAAFLVFTSCTAKNKDTHLAVDKNGKTVVERYGNLQILKNTLCDQNGNPVQLRGMSSHGLQWYGKYANNNVIRWLREDWNCQIFRTALYLGEGGFINNGSLKFLVFESIESCIKNGMYVLVDWHVLSDKDPMLYKNRAVEFFSEIAEKYGSYPNIIYEICNEPNGRNVTWDNNIKPYAEEVIATIRKYDPDNIIIVGTPNWSQDVDVAAKNPITEQKNIMYTLHFYAGSHGDKLRRKASKALKAGIPLFVTEWGTTKDSGDGGVFEKETLEWMEFLQKNNISWVNWSINNKGEDSGILKYNADKSGDGNWNEDDLSPSGIFVRKILRNEIDIK"

BASE COUNT 359 a 182 c 216 g 269 t

ORIGIN

1 atgaagaagt cctttgtatt tgtactggct gcttttttag tttttacatc atgtaccgca

61 aaaaacaagg atacccacct tgcagttgat aaaaacggaa aaaccgtagt agaacgttac

121 ggcaaccttc aaattctaaa gaacacactt tgcgaccaaa acggtaatcc cgtccagctg

181 cggggcatga gttcgcacgg tttgcaatgg tacgggaaat atgcaaataa caatgtcata

241 agatggcttc gggaggattg gaactgccaa atattccgta cagcacttta tcttggtgaa

301 ggtggtttta tcaataacgg ctcattgaaa ttcctcgttt ttgaatcaat agaatcatgt

361 attaaaaatg gtatgtatgt gcttgttgac tggcacgtgc tttctgacaa agatccgatg

421 ttgtacaaaa acagagctgt tgagtttttt tccgaaatcg ccgaaaaata cggttcatac

481 cctaatatta tctatgaaat ctgcaacgaa cccaacggaa ggaatgttac ctgggacaat

541 aacataaaac cgtatgcgga agaagtaatc gccactatca gaaaatatga tcccgacaac

601 attataatag taggaactcc aaactggagt caggacgtag atgttgccgc aaaaaatcca

661 attacagaac aaaaaaacat aatgtacaca ctgcattttt atgcaggttc gcacggggac

721 aagcttagaa gaaaagcttc gaaagcatta aaagcaggaa tccctttgtt tgtaacagaa

781 tgggggacaa ctaaagactc cggcgacggt ggtgtgtttg aaaaagaaac tttggaatgg

841 atggaattct tacagaaaaa caatatttca tgggtcaatt ggtctataaa caataaggga

901 gaagattcag gaatcttgaa atataacgcc gacaaatccg gtgatggaaa ctggaatgaa

961 gacgatttat ctccatccgg tatttttgta agaaaaattc ttcgcaatga gatagatata

1021 aaataa

- 1. **PersiCel6**

LOCUS BSeq#1 1008 bp DNA linear ENV 14-OCT-2019

ACCESSION MN821012 (will be available from 2021)

ORGANISM uncultured bacterium

Bacteria; environmental samples.

gene 1..1008

/gene="PersiCel6"

CDS 1..1008

/gene="PersiCel6"

/codon_start=1

/transl_table=11

/product="PersiCel6"

/translation="MNKKHLRAFIALFTLIFTVSCTAREAWVRVEGNKFVDPQGKELVFRGLCFSDPVKLVSDGQWNRRYFAQAAHWGANVVRFAVHPANLNAMGWEETFQAMDQGIAWAKELGMYVIMDWHSIGNLKDELYTSPMYNTTREETFKFWRTVAERYKDEPTVALYELFNEPTVTAEGVGSCSWTEWKQLQEQLIDAIRAINPHAVCLCAGFNWAYDLTPVAQEPVERPNVAYVCHPYPMKRSQPWEEQWEADFGYVADTYPVICTEIGYCLENEPGAHIPVISTDEYGEHITRYLEKKGISFTVWCFDVHWAPTLISDWDFTPTTQGRFFKAYLQEKAGK"

BASE COUNT 213 a 289 c 290 g 216 t

ORIGIN

1 atgaataaga agcatttgcg ggcttttatc gccctgttta ccctcatttt tactgtctcc

61 tgtaccgccc gggaagcctg ggtgcgcgtg gaagggaaca agtttgtgga tcctcaaggg

121 aaggaactgg tcttccgcgg cctctgtttt tcggacccgg tgaagctcgt gagcgatggc

181 cagtggaacc ggcgctattt tgcgcaggcg gcccattggg gcgccaacgt ggtccgtttc

241 gccgtccatc cggccaacct gaacgccatg ggctgggaag aaactttcca ggctatggac

301 cagggcatcg cctgggccaa agagctggga atgtatgtca ttatggactg gcattccatc

361 ggcaatctca aggacgaatt gtacacttcg cctatgtaca ataccacccg ggaggaaacg

421 ttcaagttct ggcgcaccgt ggcggagcgt tataaggacg aacccacggt agccctgtac

481 gaactgttca acgaacccac ggtgacggcc gagggcgtag gctcctgctc ctggaccgaa

541 tggaaacagt tgcaggaaca gctcatcgac gccatccgcg ccatcaatcc ccatgcggtg

601 tgcctgtgcg ccggattcaa ctgggcgtac gaccttaccc cggtggccca ggagccggtg

661 gaacgtccga atgtggctta cgtgtgccat ccttatccca tgaagcggtc ccagccctgg

721 gaggaacagt gggaggccga tttcggctat gtggccgata cctatcccgt catctgtacg

781 gaaatcggtt actgcctgga gaacgagccc ggtgcccata ttccggtaat ctccaccgat

841 gaatacggcg agcacattac ccgctacctg gagaagaagg gcatctcctt cacggtctgg

901 tgcttcgatg tccactgggc ccccacgctc atcagtgact gggatttcac tcccaccacg

961 cagggccgtt tcttcaaagc ctatcttcag gagaaggctg gaaaatag

**References**

Chen, Z., Zhao, P., Li, F., Leier, A., Marquez-Lago, T. T., Wang, Y., et al. (2018). IFeature: A Python package and web server for features extraction and selection from protein and peptide sequences. *Bioinformatics*. doi:10.1093/bioinformatics/bty140.

Chou, K. C. (2005). Using amphiphilic pseudo amino acid composition to predict enzyme subfamily classes. *Bioinformatics*. doi:10.1093/bioinformatics/bth466.

Pande, A., Patiyal, S., Lathwal, A., Arora, C., Kaur, D., Dhall, A., et al. (2019). Computing wide range of protein/peptide features from their sequence and structure. *bioRxiv*. doi:10.1101/599126.

Shen, H. B., and Chou, K. C. (2008). PseAAC: A flexible web server for generating various kinds of protein pseudo amino acid composition. *Anal. Biochem.* 373, 386–388. doi:10.1016/j.ab.2007.10.012.

Shen, J., Zhang, J., Luo, X., Zhu, W., Yu, K., Chen, K., et al. (2007). Predicting protein-protein interactions based only on sequences information. *Proc. Natl. Acad. Sci.* doi:10.1073/pnas.0607879104.

Stewart, R. D., Auffret, M. D., Warr, A., Walker, A. W., Roehe, R., and Watson, M. (2019). Compendium of 4,941 rumen metagenome-assembled genomes for rumen microbiome biology and enzyme discovery. *Nat. Biotechnol.* 37, 953–961. doi:10.1038/s41587-019-0202-3.
